# Does DeepSeek Provide Clinically Acceptable Intraocular Lens (IOL) Power Predictions in Cataract Surgery? A Proof-of-Concept Study

**DOI:** 10.3390/jcm14248870

**Published:** 2025-12-15

**Authors:** Giovanni Ottonelli, Giacomo De Rosa, Jacopo Celada Ballanti, Alessandro Gaeta, Paolo Vinciguerra, Alessandra Di Maria

**Affiliations:** 1 Department of Ophthalmology, IRCCS Humanitas Research Hospital, Rozzano, 20089 Milan, Italy; 2 Department of Internal Medicine and Medical Specialties (DIMI), Università di Genova, Viale Benedetto XV, 16132 Genova, Italy; 3 Department of Biomedical Sciences, Humanitas University, Pieve Emanuele, 20090 Milan, Italy

**Keywords:** generative artificial intelligence, artificial intelligence (AI), DeepSeek, ophthalmology, intraocular lens (IOL), spherical equivalent, biometry, cataract surgery

## Abstract

**Background/Objectives**: Accurate intraocular lens (IOL) power calculation is vital for achieving the desired postoperative spherical equivalent (SE) in cataract surgery. Generative Artificial-Intelligence (AI) systems are increasingly being used in ophthalmology to refine diagnostic and surgical planning. However, it is still unknown whether a low-cost, easily accessible generative AI model like DeepSeek can match the accuracy of conventional biometric formulas. To evaluate the accuracy of DeepSeek, an open-source generative artificial intelligence (AI), in predicting postoperative refractive spherical equivalent compared to the Barrett Universal II formula in uncomplicated cataract surgeries. **Methods**: This study analyzed biometric data from 50 eyes of 50 patients who underwent cataract surgery between July 2024 and January 2025 at Humanitas Research Hospital in Milan, Italy. Only uncomplicated cases of emmetropia with Alcon AcrySof^®^ SA60WF IOL implantation were included. 30–40 days postoperative subjective refraction was measured with a calibrated trial-frame and 6 m logMAR chart by an experienced optometrist. Prediction error (PE), median absolute error (MedAE), standard deviation (SD), and cumulative frequency of PE diopters range were calculated. A Wilcoxon signed-rank test was performed to assess statistical significance. **Results**: Barrett showed MedAE 0.36 D [0.16–0.64] and MAE 0.43 D (95% CI, 0.34–0.52) while DeepSeek-R1 showed MedAE 0.76 D [0.52–1.01] and MAE 0.77 D (95% CI, 0.67–0.87). Cumulative accuracy (AE threshold) at ±0.25/±0.50/±0.75/±1.00/±1.25/±1.50/±1.75 D was 37.7/71.7/81.1/92.5/100.0/100.0/100.0% for Barrett Universal II and 11.1/25.9/50.0/74.1/88.9/96.3/100.0% for DeepSeek-R1 (McNemar *p* < 0.01 at each threshold). The paired comparison of per-eye absolute errors favored Barrett (Wilcoxon signed-rank test, *p* < 0.0001). **Conclusions**: In this cohort, Barrett Universal II outperformed DeepSeek-R1 across MedAE/MAE and cumulative accuracy thresholds, with a significant paired difference. A general-purpose generative model used off-the-shelf (fixed A-constant, no ophthalmology-specific tuning) did not match the accuracy of a validated vergence-based formula; established formulas remain the reference standard for clinical IOL power calculation.

## 1. Introduction

Biometry in ophthalmology refers to the precise measurement of ocular anatomical features, notably corneal power and axial length, which are essential for determining the appropriate intraocular lens (IOL) power in cataract surgery [1]. By precisely capturing these variables, surgeons aim to minimize postoperative refractive errors and reduce reliance on spectacles or contact lenses [2]. Traditional ultrasound methods have largely been superseded by optical biometry techniques, such as partial coherence interferometry and low-coherence reflectometry, which allow for more accurate and reproducible AL measurements [3]. Modern IOL power formulas, including Haigis [4], Holladay 2 [5], and Barrett Universal II [6], incorporate additional parameters, such as anterior chamber depth (ACD) or lens thickness (LT), to refine predictions of the effective lens position (ELP) and give accurate results. Nonetheless, certain clinical scenarios continue to pose challenges, particularly in eyes with unusual anatomy (e.g., extreme myopia or prior refractive surgery). These situations often result in unexpected outcomes when using classical IOL formulas [7,8]. To address these outliers, advanced ray-tracing models simulate light propagation through the cornea and IOL, offering more personalized calculations [9]. Meanwhile, machine-learning approaches have started to augment or surpass conventional formulas by identifying subtle correlations in large clinical datasets [10].

For example, predictive AI aids in predicting postoperative outcomes in intraocular collamer lens (ICL) implants, refractive phacoemulsification, and cataract surgery by analyzing preoperative biometric data, helping surgeons select the optimal IOL power for better visual outcomes [11,12]. Additionally, AI approaches have been applied to complex eye biometry such as short axial length eyes [13].

On the other hand, generative artificial intelligence (AI) has emerged as a transformative tool in medicine, offering innovative solutions for diagnostics, treatment planning, and personalized care [14]. By leveraging advanced machine learning models, such as generative adversarial networks (GANs) and large language models (LLMs), generative AI can synthesize complex medical data, including imaging, genomic information, and clinical notes, to assist healthcare professionals in decision-making [15,16].

However, challenges remain, including the need for diverse datasets, data privacy concerns, and seamless integration into clinical workflows. Ethical and regulatory considerations are also critical to ensure safe and equitable use [17].

In this rapidly evolving landscape, DeepSeek has recently drawn attention as an open-source deep learning-based generative AI [18]. Since its birth in 2023, and even more since the launch of DeepSeek’s DeepThink (R1), this generative AI has been capable of advanced reasoning at lower training costs than many rivals [19,20]. Multiple research groups are now investigating how to incorporate DeepSeek within secure data environments, aiming to accelerate discovery while mitigating biases in training data [21,22].

In the context of healthcare, evidence regarding DeepSeek’s applications remains limited. This is primarily due to the very recent launch of its latest version (released on January 2025) and the competitive landscape dominated by other generative AI systems, such as ChatGPT, which have already established a significant presence in the field. While these earlier systems have been extensively studied and applied across various medical domains, ongoing research into DeepSeek may help clarify its potential healthcare applications. Specifically, we chose to evaluate DeepSeek because it is virtually unstudied in clinical contexts, thereby addressing a documented knowledge gap. Additionally, in its current R1 release, DeepSeek offers unrestricted access to the DeepThink reasoning engine and integrated web-search functions at no cost, whereas equivalent features in other generative LLMs are available only behind subscription paywalls (e.g., ChatGPT premium). To our knowledge, no study has yet assessed DeepSeek’s biometric performance as a stand-alone product nor against other generative AI models; accordingly, the present work offers an initial exploratory evaluation rather than a head-to-head comparison across platforms. We focused on ocular biometry, where AI-driven methods can refine lens constants, anticipate postoperative IOL position, and optimize IOL power selection. By utilizing DeepSeek’s advanced reasoning capabilities, we sought to evaluate its stand-alone performance as a comparable tool for standard-eye IOL power calculations. Specifically, our aim with this retrospective study is to evaluate DeepSeek’s ability to predict postoperative spherical equivalent in uncomplicated cataract surgery and to compare its performance with the Barrett Universal II formula (taken as the IOL power calculation routine gold standard).

## 2. Methods

### 2.1. Data Collection

This retrospective study analyzed 50 biometric datasets from 50 eyes of 50 patients who underwent uncomplicated cataract surgery at Humanitas Research Hospital in Milan, Italy, between July 2024 and January 2025.

To comply with contemporary IOL-power-study protocols, we performed a formal a priori sample-size calculation before data extraction [23]. Current benchmark series of uncomplicated cataract eyes report a pooled SD of the absolute error (AE) in the 0.35–0.42 D range [24]. Accepting the mid-point (SD ≈ 0.38 D) and defining a clinically meaningful difference of 0.25 D in absolute error (the threshold often used to grade “excellent” refractive outcomes), we performed an a priori sample-size calculation in G*Power 3.1 [25] with the paired-samples *t*-test as a planning approximation (two-sided α = 0.05, power = 0.80; effect size dz = 0.66). The primary analysis was prespecified as the Wilcoxon signed-rank test. The program returned a minimum requirement of 19 eyes (one eye per patient) to detect the prespecified effect. Our final cohort of 50 patients (50 eyes) therefore provides more than double the necessary information size, yielding an a priori power > 0.95 for the primary endpoint.

To ensure comparability with calculations, only patients planned for and implanted with a IOL power targeting spherical equivalent 0.00 D were included. Emmetropia was defined by choosing the first negative IOL power closest to a spherical equivalent of 0.00 diopters (D). Only eyes with CDVA ≥ 20/40 and a stable 6 m (20 ft) subjective refraction at 30–40 days were included. Postoperative CDVA ≥ 20/40 was used as an inclusion criterion to minimize potential confounding from significant posterior segment pathology or other ocular comorbidities that could affect the accuracy of refraction measurements. The exclusion criterion were a planned postoperative spherical equivalent other than emmetropia, complications during surgery and previous refractive surgery. Specifically, eyes with any history of corneal refractive surgery (LASIK, PRK, SMILE or radial keratotomy) or other corneal pathology were excluded to avoid artefactual flattening of keratometry values that could bias both vergence-formula and LLM predictions. Because the available sample (*n* = 50) would render each stratum under-powered, we did not create axial-length or keratometry subgroups. 

Preoperative biometric data were obtained using the Heidelberg Engineering Anterion^®^ biometry tool (Heidelberg, Germany) [26]. The following parameters were collected for each patient: age, gender (M/F), eye (right/left, OD/OS), keratometry values (K1 and K2, in diopters [D]), anterior chamber depth (ACD, in millimeters [mm], measured from corneal epithelium to the anterior lens surface), axial length (AL, in mm), lens thickness (LT, in mm), and white-to-white distance (WTW, in mm). Table 1 shows a summary of biometric data.

We chose the Barrett Universal II formula for its well-known overall precision compared to other formulas [24,27].

All the surgeries were performed using a temporal clear corneal main port of 2.2 mm and a 1.0 mm paracentesis. The device used for phacoemulsification was the Alcon Centurion (Fort Worth, TX, USA) and all the implanted IOLs were Alcon AcrySof^®^ SA60WF.

The final subjective manifest refraction was measured at 30–40 days postoperative with a calibrated trial-frame and 6 m logMAR chart by an experienced optometrist. The final objective refraction after cataract surgery was measured with Nidek TONOREF III. An autorefractor reading (Nidek TONOREF III, Gamagori, Japan) was recorded at the same visit for quality control; these values were not used in the primary accuracy analyses.

### 2.2. DeepSeek Prompt Design and Inference Procedure

DeepSeek-Large R1 (public build 15 January 2025) was used through the official web interface with default inference settings (temperature = 0.10, top-*p* = 1.00, max_tokens = 64). A fixed prompt template (Supplementary File S1) contained placeholders for gender, eye laterality, axial length (AL, two decimals), flat and steep keratometry (K1, K2, two decimals), anterior chamber depth (ACD, one decimal), lens thickness (LT, one decimal), white-to-white (WTW, one decimal), patient age (integer), postoperative spherical equivalent (0.00 D), and implanted IOL A-constant (119.0 for AcrySof SA60WF) was inserted in the software.

Although DeepSeek can be prompted to propose an IOL power, that figure is generated with an undisclosed, non-tunable lens constant. Implanting such a value without prior validation would conflate the model’s internal vergence assumptions with an uncontrolled constant, exposing patients to un-quantified refractive risk. To isolate and quantify that risk, we instead fixed the A-constant (119.0) and requested only the predicted postoperative spherical equivalent (SE). This prediction-error framework, recommended by contemporary IOL-formula guidelines, offers the safest and most clinically interpretable first step before considering direct implantation of model-suggested powers.

We acknowledge that the constraint of fixing the A-constant to 119.0 may have limited DeepSeek’s ability to autonomously infer optical parameters. Future work will include unconstrained testing and/or empirical A-constant calibration to more accurately assess the model’s intrinsic reasoning performance.

For each eye a new chat session was opened to avoid context carry-over. Biometric values were exported directly from the Heidelberg ANTERION^®^ database and independently cross-checked by two investigators; discrepancies or device quality-control flags triggered review of the raw scan. The validated numbers were inserted into the template, which was then submitted without additional text. The model was instructed to return a single numeric value (predicted postoperative spherical equivalent (SE) in diopters, two-decimal precision) without explanatory prose. If additional text was generated, the first number was extracted by both investigators and put into a shared spreadsheet; any discordance prompted immediate re-querying.

Repeatability was assessed on 25 eyes in triplicate; outputs were identical (coefficient of variation = 0). This confirms that the model’s outputs are reproducible and stable when provided with identical biometric data.

### 2.3. Statistical Analysis and DeepSeek Sensitivity Analysis

Postoperative subjective refraction measured 30–40 days after surgery (testing distance 6 m/20 ft) was compared with the spherical equivalent (SE) predicted by Barrett Universal II and DeepSeek in the same eyes; analyses were therefore paired and restricted to eyes with predictions from both methods. For each eye we defined absolute error (AE) (defined as the absolute value of achieved postoperative SE–predicted SE), and summarized accuracy for each method using MedAE [IQR], MAE (95% CI), and cumulative accuracy (proportion of eyes with AE ≤ 0.25/0.50/0.75/1.00/1.25/1.50/1.75 D). To compare methods inferentially, we formed the paired difference ΔAE = AE_Barrett_ − AE_DeepSeek_ and, after assessing non-normality of ΔAE with Shapiro–Wilk, used a two-sided Wilcoxon signed-rank test (α = 0.05); the Hodges–Lehmann median difference with 95% CI is reported as the effect size. Because accuracy at fixed thresholds is clinically interpretable, paired proportions at every threshold (±0.25 through ±1.75 D) were compared with McNemar’s test (two-sided, exact); at ±1.75 D both methods reached 100%, yielding no discordant pairs and a non-informative test. DeepSeek was evaluated in its publicly released configuration (fixed A-constant = 119.0, no ophthalmology-specific fine-tuning). As a complementary check, we conducted a bias-corrected sensitivity analysis: for each method we mean-centered the signed errors on the paired dataset (subtracting the method-specific cohort mean from each eye), recomputed AEs, and repeated the Wilcoxon analysis on the centered paired differences; the corresponding Hodges–Lehmann estimate, and 95% CI are also reported. After bias correction between-method differences were no longer statistically significant (HL −0.010 D, 95% CI −0.058 to 0.042; Wilcoxon *p* = 0.695), while descriptive accuracy remained comparable across thresholds. For the a priori sample-size calculation, a paired *t*-test was used as a planning approximation because it provides a straightforward and widely accepted approach to estimating the required number of observations for detecting a prespecified effect. The primary analysis, however, employed the Wilcoxon signed-rank test due to non-normality of the paired differences in absolute error (ΔAE), as determined by the Shapiro–Wilk test. The Wilcoxon test is a robust non-parametric alternative particularly when data are skewed. This ensures that the study design remains statistically valid and transparent.

### 2.4. Ethical Considerations

The protocol obtained approval from Humanitas Rozzano Ethical Committee (Protocol ID 4968, day of approval 23 September 2025) and received GDPR compliance clearance from the Data Protection Office. All biometric data were completely anonymized, and all the study phases were conducted in accordance with the Declaration of Helsinki.

## 3. Results

Data collection began in March 2025 and was completed in April 2025. As of the time of submission (June 2025), data from 50 eyes of 50 patients have been fully collected and analyzed. Statistical analyses were completed prior to submission, and findings are reported in full in the present manuscript. The following results summarize the refractive prediction performance regarding IOL power calculation of DeepSeek-R1 and Barrett Universal II.

A comparative analysis of refractive errors revealed that the Barrett Universal II formula produced a significantly lower median refractive error (MedAE 0.36 D [0.16–0.64]) and MAE 0.43 D (95% CI 0.34–0.52) compared to DeepSeek (MedAE 0.76 D [0.52–1.01] and MAE 0.77 D (95% CI 0.67–0.87)). Additionally, Barrett’s predictions spanned a narrower error range (0.015 to 1.23 D) than DeepSeek’s (0.02 to 1.70 D).

**Figure 1:**
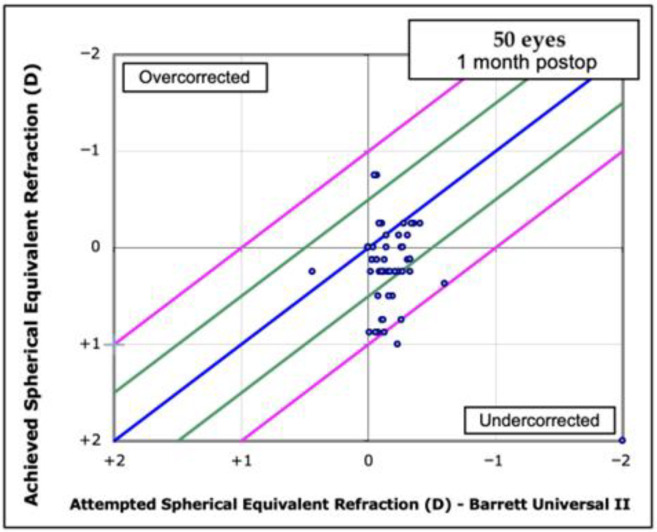
Spherical Equivalent (SEQ) Attempted vs. Achieved with Barrett II Universal formula. Attempted-vs-achieved scatter plot of attempted versus achieved spherical-equivalent refraction for 50 eyes; the blue line is the identity (y = x, zero error), while green and pink lines mark ±0.5 D and ±1.0 D error bands that frame the over- and under correction regions.

**Figure 2:**
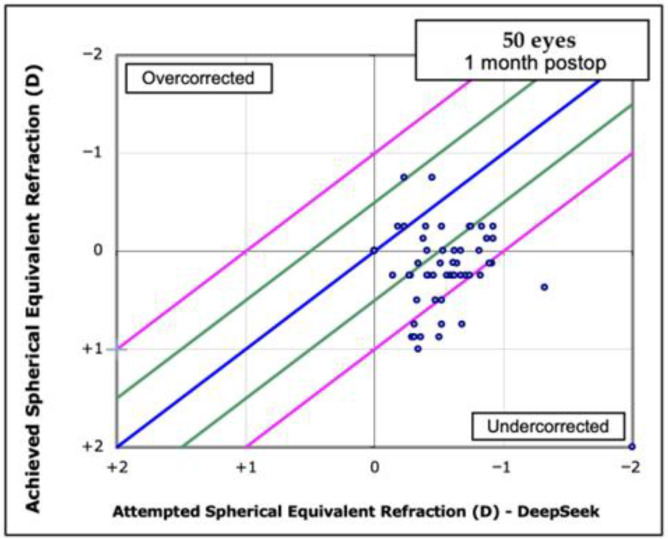
Spherical Equivalent (SEQ) Attempted vs. Achieved with DeepSeek AI. Attempted-vs-achieved scatter plot of attempted versus achieved spherical-equivalent refraction for 50 eyes; the blue line is the identity (y = x, zero error), while green and pink lines mark ±0.5 D and ±1.0 D error bands that frame the over- and under correction regions.

In Figure 1 and Figure 2, the postoperative spherical equivalent (SE) is plotted against the predicted SE for the Barrett Universal II formula and the DeepSeek method using an attempted-vs-achieved scatter plot to highlight data dispersion. Both graphs make it evident that DeepSeek systematically overestimates refractive outcomes and shows much wider scatter than Barrett, highlighting its markedly poorer performance.

DeepSeek’s points cluster below the 0-line with wider spread, showing systematic under correction and larger refractive error bands. Barrett’s points center tightly around the 0-line and stay mostly within ±0.5 D, indicating far greater accuracy and precision. The slanted blue axes make the bias clear: DeepSeek consistently overshoots the target, whereas Barrett stays balanced. Overall, Barrett outperforms DeepSeek both in mean error and in variability of postoperative refraction. Specifically, 68% of Barrett predictions lie within ±0.50 D of target compared with 22% for DeepSeek; most DeepSeek points fall below the 0-line, indicating systematic overcorrection, whereas Barrett points cluster tightly around zero within the ±0.5 D and ±1.0 D boundaries.

**Figure 3:**
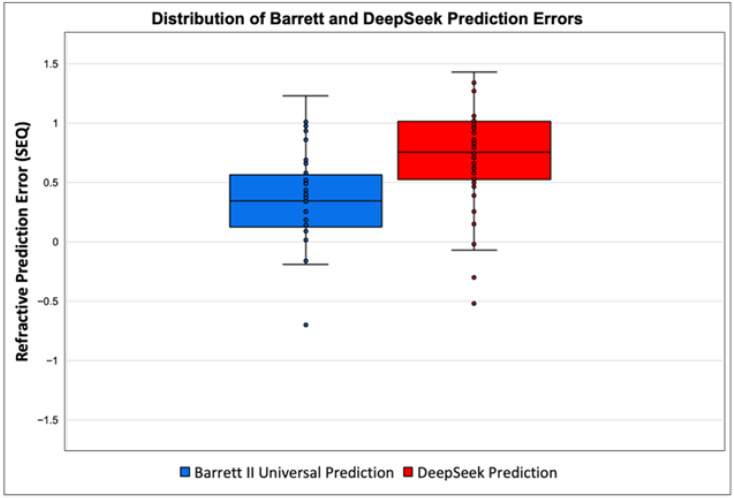
Box plot of spherical-equivalent prediction errors comparing Barrett Universal II and DeepSeek AI; Barrett has a lower median error and narrower spread, while DeepSeek consistently overestimates refraction.

Figure 3 provides further insight into data distributions through box plots. This graphical analysis indicated a clear difference between median absolute refractive errors and interquartile ranges of the two methods, highlighting more frequent and larger errors in predictions generated by DeepSeek. A Wilcoxon signed-rank test on absolute prediction errors (APE) confirmed that this difference was statistically highly significant (*p* < 0.0001). As illustrated in Figure 3, DeepSeek had a higher median absolute error (MedAE = 0.755 D) than Barrett (MedAE = 0.36 D); Barrett’s IQR was 0.50 D (0.10–0.60 D) versus DeepSeek’s 0.40 D (0.50–0.90 D), yet DeepSeek displayed a wider overall range (0.02–1.70 D vs. 0.015–1.23 D), with only a few mild negative outliers (<−0.5 D) in both groups.

**Figure 4:**
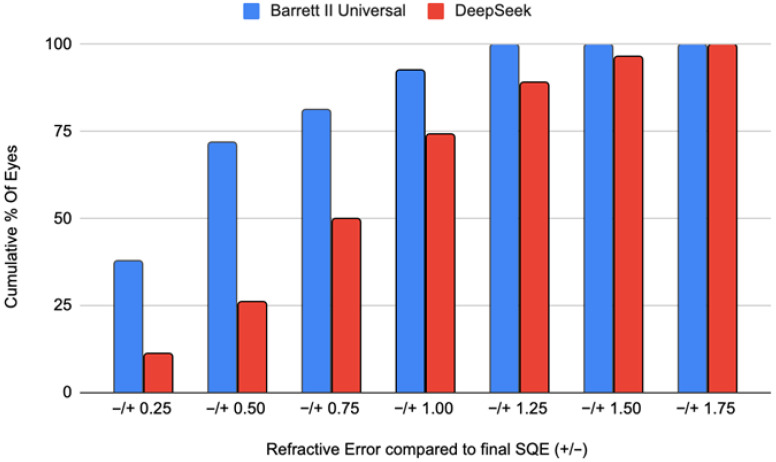
Cumulative Frequency. Cumulative frequency of eyes within successive spherical-equivalent error thresholds for Barrett Universal II and DeepSeek AI, showing Barrett’s higher accuracy up to ±1.0 D before the curves converge.

The cumulative frequency of prediction errors shown in Figure 4 further underscores the Barrett Universal II formula’s superior accuracy across all spherical equivalent thresholds examined. Cumulative accuracy favored Barrett at across all explored thresholds (0.25/0.50/0.75/1.00/1.25/1.50/1.75) achieving 37.7/71.7/81.1/92.5/100.0/100.0/100.0%, respectively, whereas DeepSeek achieved 11.1/25.9/50.0/74.1/88.9/96.3/100.0% (McNemar *p* < 0.01 at each threshold).

Potential covariates influencing prediction accuracy were evaluated. Spearman correlation analysis revealed no significant association between axial length and refractive prediction error for either Barrett (ρ = 0.05, *p* = 0.73) or DeepSeek (ρ = −0.01, *p* = 0.94). This indicates that variations in axial length, including cases of high myopia or hyperopia, did not meaningfully influence the accuracy of refractive predictions for either formula. Similarly, Spearman correlations between per-eye AE and K1/K2/ACD/LT were not statistically significant. Barrett demonstrated a weak and non-significant positive correlation (ρ = 0.12, *p* = 0.41), while DeepSeek showed a slightly stronger, but still non-significant, correlation (ρ = 0.17, *p* = 0.24), suggesting that anterior corneal curvature had no measurable effect on predictive accuracy. K2 values showed a comparable trend. For Barrett, the correlation was weak (ρ = 0.07, *p* = 0.62), and for DeepSeek, similarly low and non-significant (ρ = 0.12, *p* = 0.43). This indicates that vertical corneal steepness or flatness did not significantly affect refractive prediction errors. Anterior chamber depth (ACD) also showed no significant correlation with MedAE. The correlation coefficients were negligible for both Barrett (ρ = −0.01, *p* = 0.93) and DeepSeek (ρ = −0.07, *p* = 0.63), implying that the anatomical variation in ACD had no discernible impact on model performance. Lastly, lens thickness (LT) did not correlate significantly with prediction errors in either method. Barrett yielded ρ = 0.09 (*p* = 0.54), and DeepSeek ρ = 0.04 (*p* = 0.77), further supporting that biometric variability in LT does not play a relevant role in the refractive prediction accuracy of these models.

In summary, these expanded analyses demonstrate that DeepSeek did not achieve comparable accuracy compared to the Barrett Universal II formula, consistently showing higher median refractive error, significant systematic bias, and lower cumulative accuracy, independent of axial length or refractive extremes in this patient cohort.

### Subgroup Analysis: Eyes with DeepSeek-R1 Absolute Prediction Error > 1.0 D

Among the 50 analyzed cases, 13 eyes (26%) exhibited an absolute DeepSeek-R1 prediction error greater than ±1.0 D. To investigate DeepSeek’s performance more deeply, we further analyzed these 13 cases to find some correlation with biometric data and correlate them with that of the remaining 37 study eyes (shown in detailed in Table 2 and Table 3). Eyes in the high-error subgroup were only marginally shorter (axial length 23.42 ± 0.78 mm versus 23.76 ± 1.28 mm) and displayed virtually the same corneal power (mean keratometry 43.52 ± 0.66 D versus 43.46 ± 1.50 D). Anterior chamber depth was slightly shallower (2.97 ± 0.39 mm versus 3.17 ± 0.45 mm), lens thickness modestly greater (4.98 ± 0.35 mm versus 4.52 ± 0.45 mm) and horizontal corneal diameter marginally smaller (11.89 ± 0.28 mm versus 11.98 ± 0.36 mm), yet none of these differences was significant on a two-tailed Welch’s *t*-test (all *p* > 0.10; findings were unchanged when re-checked with a Mann–Whitney test). Because the largest DeepSeek discrepancies occurred within an otherwise routine biometric range, they appear to reflect limitations in the model’s untreated lens-position estimation rather than simple extrapolation error at anatomical extremes, underscoring the need for ophthalmology-specific fine-tuning or external calibration before clinical deployment.

## 4. Discussion

Our findings indicate that DeepSeek, as an open-source generative AI, demonstrated significantly lower accuracy in IOL power prediction compared to the Barrett Universal II formula. The median absolute error (MedAE) for DeepSeek was markedly higher (0.755 D vs. 0.36 D), with a statistically significant difference (*p* < 0.001). These results, in the specific context of standard IOL power calculation targeting spherical equivalent 0.00 D, demonstrate that DeepSeek did not achieve the level of predictive accuracy required for clinical reliability. It is important however to state that these findings should not be interpreted as a general statement on the adequacy of generative AI in ophthalmology but rather reflect its current limitations in this narrowly defined application.

Previous studies have highlighted the potential of AI in ophthalmology, particularly in diagnostic applications and decision-support tools for complex biometric cases [6,17]. However, large language models such as DeepSeek require extensive domain-specific fine-tuning to meet the rigorous accuracy standards necessary for clinical application [14]. The performance gap observed in this study suggests that DeepSeek lacks the precision required to replace established biometric formulas, particularly in standard IOL calculations. Recent studies have further emphasized the importance of validating AI models through rigorous clinical testing and fine-tuning, particularly when incorporating AI into systems like IOL calculations in cataract surgery [28].

Furthermore, our study underscores the importance of validated, human-supervised methodologies in AI integration for medical applications. Cumulative accuracy favored Barrett at ±0.25/±0.50/±1.00 D (37.7/71.7/92.5%) vs. DeepSeek (11.1/25.9/74.1%), consistent with McNemar paired comparisons. This trend is consistent with prior research emphasizing the robustness of traditional biometric formulas over purely AI-driven approaches [7]. Several studies have reinforced the need for a human-centered, collaborative approach when applying AI in ophthalmology, ensuring AI models remain adjunctive tools rather than independent decision-makers. AI should function as a support tool for human expertise, such as in the management of complex cases or rare diseases [29]. A potential explanation for DeepSeek’s underperformance lies in its inherent training limitations. Unlike established formulas optimized for ocular biometric calculations, DeepSeek is a generalist AI model not specifically trained on ophthalmic datasets. Additionally, generative AI may introduce biases due to dataset selection and input variability, which could further compromise its accuracy in a clinical setting [30]. The application of AI in ophthalmology, especially for disease prediction and management, is highly reliant on training data quality. Current AI models, if not properly trained with domain-specific data, may fail to meet the precision required for clinical practice. This highlights the importance of training AI with large, accurate datasets that are specifically curated for ophthalmic diseases [31].

Interestingly, DeepSeek’s development highlights a broader shift in the AI landscape. The model’s efficiency in reasoning and problem-solving, despite using fewer computational resources than its competitors, signals a potential paradigm shift away from the traditional scaling law of AI, which prioritizes larger datasets and increased computational power [20]. The rapid development of DeepSeek R1, achieved with a small research team, has demonstrated that innovative algorithmic strategies can match or even surpass larger, more resource-intensive models [15]. Such innovations in AI algorithms point to the growing potential of AI to revolutionize medical practices, including ophthalmology, through more efficient, scalable tools that are less dependent on resource-intensive infrastructures [32]. Furthermore, DeepSeek’s implementation of Mixture of Experts, reinforcement learning, and FP8 mixed precision frameworks has allowed it to achieve notable efficiency gains while maintaining competitive performance across benchmarks [9]. In ophthalmology, AI models like DeepSeek could potentially aid in predicting systemic parameters, helping doctors not only assess eye health but also detect early signs of systemic diseases that may manifest through ophthalmic imaging [33].

A recent preprint by Mikhail et al. described the application of DeepSeek-R1 in a simulated ophthalmology setting, evaluating its diagnostic and management reasoning across 300 ophthalmology case-based scenarios spanning ten subspecialties using StatPearls content. DeepSeek-R1 achieved comparable overall accuracy (~82%) to OpenAI o1 while reducing cost per query by an estimated 15-fold, demonstrating high performance and reduced cost [34].

Regarding specific comparison with other generative-AI models in the field of IOL calculation we failed to produce evidence. To date, no peer-reviewed publications have evaluated generative large language models (including ChatGPT, ARI, or other LLM systems) for the narrow and specific task of IOL calculation; consequently, it is not yet possible to perform a formal comparative analysis between these models and DeepSeek. As such, our findings are limited to the performance of DeepSeek under these narrowly defined conditions, and no inference can yet be made about the relative performance of other LLMs in this context.

Another distinguishing feature of DeepSeek is its reasoning process. Unlike traditional LLMs, which often function as black boxes, DeepSeek leverages chain-of-thought (CoT) reasoning to break down complex tasks into logical steps. It should be noted that chain-of-thought (CoT) reasoning is a feature shared by many large language models and is not unique to DeepSeek. Similarly, the “black-box” nature of model predictions remains a fundamental limitation across generative AI systems. Acknowledging these aspects provides a balanced perspective on the interpretability and safety considerations when applying such models in clinical settings. This characteristic could theoretically benefit medical applications, such as differential diagnoses, where multi-step reasoning is essential [12]. However, in our study, this reasoning approach did not translate into improved accuracy for biometric predictions, suggesting that domain-specific adaptations are still required for effective integration in ophthalmology.

Specifically, DeepSeek could be fine-tuned using lightweight domain-adaptation techniques (e.g., LoRA) on curated biometric datasets paired with postoperative refractive outcomes to enhance calibration and task-specific accuracy [35]. Furthermore, a hybrid residual model could be designed where DeepSeek’s output is refined by a secondary adjustment layer trained on the residuals of a validated IOL formula such as Barrett Universal II, merging generative reasoning with established optical calculation frameworks [36]. Finally, embedding such a system into preoperative planning platforms (where both AI-generated and formula-based predictions are displayed side by side for surgeon review) would enable iterative calibration and safeguard clinical reliability, in line with prior machine learning models that incorporate ocular biometric parameters to estimate postoperative lens position [37].

### Limitations

While our study provides valuable insights into the performance of DeepSeek in IOL power prediction, several limitations should be acknowledged mainly due to methodological choices.

First, we exclusively analyzed cases targeting spherical equivalent 0.00 D, which may limit the generalizability of our findings to other targets, such as myopic spherical equivalent.

From a methodological perspective, this restriction was not strictly mandated by the way our error metrics are defined and should be regarded as a pragmatic simplification for this initial proof-of-concept analysis, a choice that inevitably reduced the effective sample size, attenuated statistical power, and limited our ability to explore model performance across different refractive targets and axial-length entries.

Additionally, preoperative refraction was not included as a parameter, potentially reducing the ability to fully understand individual variability in postoperative outcomes. Our study also lacked a detailed longitudinal assessment of postoperative refractive trends, which could have provided further insight into the long-term predictive accuracy of the AI model.

Another significant limitation is the exclusion of eyes with extreme biometric values, such as those with high myopia or hyperopia, limiting applicability to real-world clinical populations where predictive performance might differ, particularly in challenging biometric profiles.

The cohort, therefore lacked sufficiently large subsets of extremely long, short or flat corneas to permit robust subgroup analysis. Although our evaluation was confined to eyes with regular anatomy targeting spherical equivalent 0.00 D, surgeons routinely encounter more complex scenarios (e.g., long axial length, high myopia, prior keratorefractive surgery) where even state-of-the-art vergence formulas show a measurable drop in predictive accuracy. Recent evidence demonstrates that small discrepancies in the way axial-length adjustments are handled can translate into clinically relevant refractive surprises in these extreme eyes [38]. The selected cohort largely concentrated around normal axial length values, with relatively few cases representing short (AL less than 22 mm) or long eyes (AL more than 26.5 mm). Although this criterion was chosen to minimize confounding factors and standardize the analysis in this preliminary investigation, it unintentionally produced a non-representative sample that does not reflect the typical biometric distribution encountered in clinical practice. This imbalance introduces a selection bias that may limit the generalizability of our findings and may not fully capture DeepSeek’s performance in eyes at the extremes of the axial-length spectrum, where IOL power calculations are most challenging. Recognizing this limitation, future studies will deliberately incorporate a broader and more evenly distributed range of biometric values to provide a more comprehensive evaluation of the model across diverse clinical scenarios. Accordingly, DeepSeek’s accuracy in eyes with extreme biometry is likely inferior to what we observed.

These were deliberate choices to reduce confounding effects associated with extreme biometric values and to ensure a homogeneous sample for this initial evaluation. By doing so we prioritized internal consistency and control of confounders, but this could potentially lead to selection bias.

Another key limitation of this study is that DeepSeek was evaluated “off the shelf,” without domain-specific fine-tuning on biometric data or calibration of lens-specific constants. This choice reflects the current state of the platform: the model does not yet expose an interface for supervised retraining, nor does it provide a parameter analogous to the A-constant used in vergence formulas. Constant calibration remains a cornerstone of modern IOL power calculation. Barrett Universal II was run with our institution’s optimized lens constant, whereas DeepSeek predictions used a user-specified A-constant (119.0). This asymmetry may introduce an additive bias unrelated to model performance; accordingly, between-method comparisons should be interpreted with caution and we emphasize absolute-error metrics (MAE/median AE) over mean error. Even in anatomically routine eyes, small A-constant refinements (often <0.10 D) translate almost one-to-one into postoperative refractive error, making them clinically important. A recent prospective series showed that performing even a limited constant calibration reduced mean prediction error by nearly 0.10 D in otherwise uncomplicated cases [39]. Because DeepSeek currently delivers a fixed, non-tunable estimate, it cannot benefit from this optimization step; this constraint likely contributed to the systematic bias we observed and will continue to limit the model’s precision until a mechanism for lens-constant adjustment is implemented. In addition, the lack of IOL constant optimization for DeepSeek predictions, the small sample size, and the absence of a validation cohort further limit the generalizability of the findings and increase the risk of model overfitting

A potential limitation of this study relates to the handling of AI model interactions. Although each eye was analyzed in a separate session to minimize short-term cross-case contamination, we did not have access to verified information regarding long-term data logging, retention, or learning-contamination control by the model developer. Consequently, it cannot be fully excluded that previous inputs may have influenced subsequent predictions. Future studies could address this issue by using paid APIs with confirmed no-logging policies or isolated local deployments to ensure complete data independence.

Although we performed a subgroup analysis of eyes with DeepSeek-R1 absolute prediction error > 1.0 D, we did not report the corresponding Barrett Universal II errors for these cases. Including this comparison would help clarify whether large errors reflect limitations of the generative model or intrinsically challenging eyes with unmodeled biometric complexity. Future studies will incorporate this comparative analysis to provide a more complete understanding of DeepSeek’s performance in outlier cases.

Lastly, technical challenges, including frequent server congestion, resulted in slow data retrieval, with some calculations taking up to a week. This raises concerns regarding the practicality of real-time clinical implementation. In addition, the lack of IOL constant optimization for DeepSeek predictions, the small sample size, and the absence of a validation cohort further limit the generalizability of the findings. Future studies should address these challenges by refining generative AI methodologies and incorporating a wider range of biometric profiles.

## 5. Conclusions

Despite its methodological limitations this represents a first attempt to explore generative-AI in the narrow and specific field of IOL power calculations. Our results suggest that, at present, DeepSeek-R1 in its current form has not achieved comparable results compared to standardized methods (Barrett II Universal) for IOL calculation in ophthalmic surgery.

Prospective studies should explore AI fine-tuning techniques tailored to ophthalmology, ensuring data governance and compliance with clinical safety standards [19]. As AI continues to evolve in ophthalmology, the focus must shift toward refining its integration with existing clinical practices and ensuring that AI-driven tools remain compatible with medical ethics and clinical governance [40].

Despite its limitations, generative AI still holds promise as an adjunct tool rather than a standalone solution. Future research should focus on integrating AI with traditional biometric formulas to harness the pattern recognition strengths of deep learning while maintaining the reliability of validated formulas.

In conclusion, while generative AI represents a promising area of innovation, its use in IOL power prediction requires further development and ophthalmology-specific validation. Our results apply specifically to standard IOL calculations in emmetropic eyes, and broader conclusions about its clinical readiness in other domains of ophthalmology should be drawn cautiously. We hope this could represent a first step in the integration of generative-AI models in the IOL calculation path.

### Value Statement

What was known:

- AI has been increasingly integrated into biometric formulas to enhance IOL power calculations.
- Traditional formulas, such as Barrett Universal II, have demonstrated high accuracy in predicting postoperative refractive outcomes.
- The accuracy and reliability of generative AI models, such as DeepSeek, for biometric calculations remain unproven.

What this paper adds:

- DeepSeek demonstrated significantly lower accuracy in IOL power prediction compared to the Barrett Universal II formula.
- Generative AI models currently fail to achieve comparable accuracy in biometric calculations, with higher mean refractive errors and greater variability.
- Despite AI’s advancements, human-validated formulas remain essential for achieving optimal refractive outcomes in cataract surgery.

## Figures and Tables

**Table 1:** Biometric data demographics.

	Mean ± SD	Minimum	Maximum
AXL (mm)	23.67 ± 1.17	22.12	29.30
K1 (D)	42.88 ± 1.37	37.90	45.55
K2 (D)	44.06 ± 1.29	40.20	46.37
ACD (mm)	3.12 ± 0.44	2.41	4.90
LT (mm)	4.64 ± 0.47	3.40	5.56
WTW (mm)	11.96 ± 0.34	11.20	12.80

The table shows the mean and standard deviation (SD), the minimum and the maximum value of the reported parameters. AXL = axial length; K1 = flat keratometry; K2 = steep keratometry; ACD = anterior chamber depth; LT = lens thickness; WTW = white-to-white.

**Table 2:** Pre-operative biometry of eyes with large DeepSeek error (>1.0 D) versus the remainder of the cohort. Values are mean ± SD. Differences between groups were assessed with a two-tailed Welch’s *t*-test (all *p* > 0.10; results unchanged on Mann–Whitney re-check). ACD = anterior chamber depth; WTW = white-to-white corneal diameter.

Parameter	>1.0 D Error (*n* = 13)	≤1.0 D Error (*n* = 37)
**Axial length (mm)**	23.42 ± 0.78	23.76 ± 1.28
**K1 (D)**	42.99 ± 0.69	42.85 ± 1.55
**K2 (D)**	44.04 ± 0.63	44.06 ± 1.46
**ACD (mm)**	2.97 ± 0.39	3.17 ± 0.45
**Lens thickness (mm)**	4.98 ± 0.35	4.52 ± 0.45
**WTW (mm)**	11.89 ± 0.28	11.98 ± 0.36

**Table 3:** The implanted IOL power showed a mean value of 21.40 ± 1.87 D, spanning from 16.00 to 26.00 D. The postoperative spherical equivalent averaged 0.18 ± 0.40 D, with a range between −0.75 and +1.00 D.

	Mean ± SD	Minimum	Maximum
**Implanted IOL (D)**	21.40 ± 1.87	16.00	26.00
**Postoperative Spherical Equivalent (D)**	0.18 ± 0.40	−0.75	1.00

## Data Availability

The de-identified per-eye dataset is available from the corresponding author upon reasonable request; it is not publicly shared to safeguard participant privacy.

## Supplementary Materials

The following supporting information can be downloaded at: https://www.mdpi.com/article/10.3390/jcm14248870/s1, File S1: DeepSeek Prompt Template; File S2: full database.
