# Supplementary material for: Does DeepSeek Provide Clinically Acceptable Intraocular Lens (IOL) Power Predictions in Cataract Surgery? A Proof-of-Concept Study
Source: J Clin Med. 2025 Dec 15;14(24):8870. doi: 10.3390/jcm14248870 (PMC12733728; doi:10.3390/jcm14248870)
Supplement: Supplementary file 1 [file jcm-14-08870-s001.zip › Supplementary File S1.pdf]

Supplementary File S1 – DeepSeek Prompt Template

Model version: DeepSeek-Large R1 (public build 2025-01-15)

Inference settings: temperature 0.10 │ top-p 1.00 │ max_tokens 64

Paste the text below into a new DeepSeek chat session for each eye and replace the bracketed placeholders with the validated numeric values. Do NOT add any additional text when submitting the prompt.

Please predict the postoperative spherical equivalent (SE) in dioptres, to one-decimal precision, for the following cataract case after implantation of an Alcon AcrySof SA60WF IOL (A-constant 119.0).

Return ONLY the numeric SE value.

• Gender (Male/Female): [GENDER]

• Eye (OD/OS): [EYE]

• Axial length (AL): [AL_mm]

• Flat keratometry (K1): [K1_D]

• Steep keratometry (K2): [K2_D]

• Anterior-chamber depth (ACD): [ACD_mm]

• Lens thickness (LT): [LT_mm]

• White-to-white (WTW): [WTW_mm]

• Patient age: [AGE_Y]

• Target refraction: 0.00 D

Example (placeholders filled):

• Gender (Male/Female): Male

• Eye (OD/OS): OD

• Axial length (AL): 24.87 mm

• Flat keratometry (K1): 44.31 D

• Steep keratometry (K2): 45.12 D

• ACD: 3.46 mm

• LT: 4.45 mm

• WTW: 12.1 mm

• Patient age: 72 years

• Target refraction: 0.00 D
