# Supplementary material for: Does DeepSeek Provide Clinically Acceptable Intraocular Lens (IOL) Power Predictions in Cataract Surgery? A Proof-of-Concept Study
Source: J Clin Med. 2025 Dec 15;14(24):8870. doi: 10.3390/jcm14248870 (PMC12733728; doi:10.3390/jcm14248870)
Supplement: Supplementary file 1 [file jcm-14-08870-s001.zip › Supplementary File S2.pdf]

**File S2 – full database***

| N° | sex (M=1; F=0) | Eye | AXL | K1 | K2 | ACD | LT | WTW | IOL (Barrett) | SE target (Barrett) | SE target (Deepseek) | Spheric Equivalent Subjective | Barrett MRE | DeepSeek MRE |
|---|---|---|---|---|---|---|---|---|---|---|---|---|---|---|
| 1 | 1 | OD | 24.71 | 41.77 | 42.68 | 3.48 | 4.40 | 11.9 | 19.5 | -0.26 | -0.68 | 0.75 | 1.01 | 1.43 |
| 2 | 0 | OD | 24.27 | 43.77 | 44.94 | 3.19 | 5.00 | 11.8 | 18 | -0.12 | -0.82 | 0.25 | 0.37 | 1.07 |
| 3 | 0 | OD | 23.29 | 42.32 | 43.1 | 3.49 | 4.78 | 12.3 | 23.5 | -0.11 | -0.56 | 0.25 | 0.36 | 0.81 |
| 4 | 0 | OD | 24.44 | 42.95 | 44.55 | 3.99 | 4.59 | 12.7 | 18.5 | -0.27 | -0.81 | 0 | 0.27 | 0.81 |
| 5 | 1 | OD | 26.96 | 39 | 40.7 | 4.06 | 3.7 | 12.0 | 19 | -0.28 | -0.83 | -0.25 | 0.03 | 0.58 |
| 6 | 1 | OD | 23.81 | 43.41 | 44.58 | 3.67 | 4.29 | 11.7 | 20 | -0.07 | -0.64 | 0.125 | 0.195 | 0.765 |
| 7 | 0 | OD | 22.54 | 43.45 | 44.33 | 2.99 | 4.82 | 12.1 | 24.5 | -0.31 | -0.91 | 0.125 | 0.435 | 1.035 |
| 8 | 1 | OD | 23.06 | 42.34 | 43.12 | 2.84 | 4.37 | 12.0 | 23.5 | -0.11 | -0.52 | -0.25 | -0.14 | 0.27 |
| 9 | 1 | OD | 23.36 | 43.26 | 44.53 | 3.12 | 5.02 | 12.3 | 22 | -0.08 | -0.36 | 0.875 | 0.955 | 1.235 |
| 10 | 0 | OD | 22.75 | 44.93 | 45.89 | 3.26 | 3.87 | 11.7 | 21.5 | -0.09 | -0.59 | 0.25 | 0.34 | 0.84 |
| 11 | 1 | OD | 29.3 | 37.9 | 40.2 | 3.46 | 3.8 | 11.9 | 16 | -0.31 | -0.92 | -0.125 | 0.185 | 0.795 |
| 12 | 1 | OD | 23.35 | 43.2 | 44.09 | 2.65 | 5.03 | 12.01 | 22 | -0.33 | -0.42 | 0.25 | 0.58 | 0.67 |
| 13 | 1 | OD | 23.61 | 42.94 | 44.15 | 3.18 | 4.57 | 11.9 | 21.5 | -0.07 | -0.45 | -0.75 | -0.68 | -0.3 |
| 14 | 0 | OD | 23.63 | 42.57 | 43.45 | 2.91 | 4.77 | 12.2 | 21.5 | -0.09 | -0.4 | -0.25 | -0.16 | 0.15 |
| 15 | 0 | OD | 22.96 | 43.32 | 44.87 | 3.03 | 4.46 | 12.2 | 23 | -0.36 | -0.23 | -0.25 | 0.11 | -0.02 |
| 16 | 1 | OD | 22.64 | 43.56 | 44.53 | 2.50 | 5.15 | 11.3 | 23.5 | -0.13 | -0.5 | 0.875 | 1.005 | 1.375 |
| 17 | 0 | OD | 23.75 | 43.13 | 43.54 | 3.55 | 4.57 | 11.9 | 21 | -0.19 | -0.52 | 0.50 | 0.69 | 1.02 |
| 18 | 1 | OD | 23.44 | 43.56 | 45.35 | 3.32 | 4.65 | 12.4 | 21 | -0.1 | -0.28 | 0.25 | 0.35 | 0.53 |
| 19 | 0 | OD | 23.77 | 42.29 | 43.39 | 3.08 | 4.98 | 12.3 | 22 | -0.09 | -0.18 | -0.25 | -0.16 | -0.07 |
| 20 | 0 | OD | 23.29 | 45.55 | 46.37 | 3.33 | 4.37 | 11.8 | 19.5 | -0.24 | -0.14 | 0.25 | 0.49 | 0.39 |
| 21 | 1 | OD | 23.14 | 43.61 | 44.43 | 2.68 | 5.44 | 11.7 | 22 | -0.06 | -0.29 | 0.875 | 0.935 | 1.165 |
| 22 | 1 | OD | 24 | 42.65 | 43.54 | 2.78 | 5.05 | 12.1 | 20.5 | -0.23 | -0.34 | 1.0 | 1.23 | 1.34 |
| 23 | 1 | OD | 23.91 | 42.37 | 44.28 | 3.48 | 4.89 | 12 | 20.5 | -0.01 | -0.31 | 0.875 | 0.885 | 1.185 |
| 24 | 0 | OD | 23.75 | 41.83 | 43.48 | 2.75 | 4.85 | 12 | 21.5 | -0.08 | -0.33 | 0.50 | 0.58 | 0.83 |
| 25 | 1 | OD | 23.15 | 43.38 | 45.37 | 3.71 | 4.08 | 12.4 | 22.5 | -0.12 | -0.41 | 0.25 | 0.37 | 0.66 |
| 26 | 1 | OD | 24.97 | 41.07 | 42.64 | 3.30 | 4.80 | 12.8 | 19 | -0.09 | -0.74 | -0.25 | -0.16 | 0.49 |

| | | | | | | | | | | | | | | |
|---|---|---|---|---|---|---|---|---|---|---|---|---|---|---|
| 27 | 0 | OD | 22.63 | 43.52 | 44.59 | 2.72 | 4.94 | 12.4 | 24 | -0.24 | -0.87 | -0.125 | 0.115 | 0.745 |
| 28 | 0 | OD | 22.14 | 42.35 | 43.38 | 2.85 | 4.48 | 12.2 | 26 | -0.6 | -1.32 | 0.375 | 0.975 | 1.695 |
| 29 | 0 | OD | 23.65 | 43.22 | 44.10 | 2.51 | 5.32 | 12 | 20.5 | -0.11 | -0.52 | 0.75 | 0.86 | 1.27 |
| 30 | 0 | OD | 23.75 | 41.5 | 42.78 | 2.65 | 5.15 | 12 | 22 | -0.05 | -0.23 | -0.75 | -0.7 | -0.52 |
| 31 | 1 | OD | 23.89 | 41.96 | 43.71 | 3.03 | 5 | 11.5 | 21 | -0.12 | -0.31 | 0.75 | 0.87 | 1.06 |
| 32 | 1 | OD | 24.21 | 41.5 | 42.74 | 3.11 | 5.26 | 11.7 | 21 | -0.15 | -0.27 | 0.25 | 0.4 | 0.52 |
| 33 | 0 | OD | 23 | 43.8 | 45.3 | 3.22 | 4.23 | 11.8 | 22 | -0.16 | -0.47 | 0.5 | 0.66 | 0.97 |
| 34 | 0 | OD | 23.87 | 43.9 | 44.84 | 3.48 | 4.44 | 12 | 19.5 | -0.21 | -0.71 | 0.25 | 0.46 | 0.96 |
| 35 | 0 | OD | 23.46 | 43.01 | 44.73 | 3.04 | 4.39 | 12.0 | 21 | -0.03 | -0.34 | 0.125 | 0.155 | 0.465 |
| 36 | 0 | OD | 23.54 | 42.67 | 43.89 | 2.97 | 4.58 | 11.3 | 21.5 | -0.14 | -0.38 | -0.125 | 0.015 | 0.255 |
| 37 | 0 | OD | 23.49 | 42.75 | 43.71 | 3.30 | 4.93 | 12.5 | 22 | -0.02 | -0.46 | 0.25 | 0.27 | 0.71 |
| 38 | 0 | OD | 22.71 | 43.7 | 44.76 | 2.63 | 4.29 | 11.8 | 23 | -0.14 | -0.53 | 0 | 0.14 | 0.53 |
| 39 | 0 | OD | 23.01 | 44.88 | 46.04 | 3.44 | 4.60 | 11.5 | 21 | -0.04 | -0.62 | 0 | 0.04 | 0.62 |
| 40 | 1 | OD | 23.70 | 43.47 | 44.29 | 2.90 | 3.92 | 11.4 | 20 | -0.11 | -0.67 | 0.25 | 0.36 | 0.92 |
| 41 | 1 | OD | 22.49 | 43.78 | 44.54 | 2.41 | 5.56 | 11.8 | 24 | -0.03 | -0.89 | 0.125 | 0.155 | 1.015 |
| 42 | 0 | OD | 22.52 | 43.79 | 45.05 | 2.76 | 5.05 | 11.5 | 23.5 | -0.13 | -0.51 | 0.125 | 0.255 | 0.635 |
| 43 | 1 | OD | 22.12 | 43.16 | 44.9 | 2.56 | 4.55 | 12 | 25 | -0.34 | -0.92 | -0.25 | 0.09 | 0.67 |
| 44 | 0 | OD | 23 | 44 | 44.5 | 3.39 | 3.83 | 12.3 | 21.5 | 0.44 | -0.61 | 0.25 | -0.19 | 0.86 |
| 45 | 0 | OD | 25.06 | 40.3 | 40.7 | 4.36 | 3.4 | 11.9 | 21 | -0.41 | -0.75 | -0.25 | 0.16 | 0.5 |
| 46 | 0 | OD | 25.08 | 41.7 | 42.6 | 4.9 | 4.2 | 11.7 | 21.5 | -0.17 | -0.74 | 0.25 | 0.42 | 0.99 |
| 47 | 1 | OD | 23.84 | 42.19 | 43.45 | 3.24 | 4.75 | 12.1 | 21.5 | -0.26 | -0.41 | 0 | 0.26 | 0.41 |
| 48 | 0 | OD | 23.85 | 44.29 | 45.33 | 3.03 | 5.29 | 11.2 | 19 | -0.27 | -0.67 | 0 | 0.27 | 0.67 |
| 49 | 0 | OD | 23.68 | 43.96 | 44.84 | 3.09 | 4.59 | 11.8 | 20 | -0.27 | -0.62 | 0.25 | 0.52 | 0.87 |
| 50 | 0 | OD | 22.98 | 44.72 | 46 | 3.00 | 4.88 | 12.0 | 21.5 | -0.33 | -0.61 | 0.12 | 0.45 | 0.73 |

***Dataset fully anonymized according to GDPR and Helsinki.**
